# Evaluation of bony fusion after anterior cervical discectomy: a systematic literature review and meta-analysis

**DOI:** 10.1007/s10143-025-03542-w

**Published:** 2025-04-25

**Authors:** Floor E. de Vries, Azra Gül, Ignacio Mesina-Estarrón, Rania A. Mekary, Carmen L. A. Vleggeert-Lankamp

**Affiliations:** 1 Department of Neurosurgery, Leiden University Medical Center (LUMC), Albinusdreef 2, Leiden, 2333 ZA The Netherlands; 2 Computational Neuroscience Outcomes Center at Harvard, Department of Neurosurgery, Brigham and Women’s Hospital, 75 Francis St, Boston, MA 02115 USA; 3 Harvard Medical School, 25 Shattuck St, Boston, MA 02115 USA; 4 Department of Pharmaceutical Business and Administrative Sciences, School of Pharmacy, MCPHS University, 179 Longwood Ave, Boston, MA 02115 USA

**Keywords:** Bony fusion, Fusion rate, Fusion assessment, Anterior cervical discectomy, Cervical spine, Fusion timing

## Abstract

Significant variability exists in reported fusion rates in the cervical spine after anterior discectomy. Here we review fusion assessment methods, timing of fusion with various intervertebral devices, and examine correlations with clinical outcomes. PubMed, Medline, Embase, Web of Science, Cochrane Library, and Emcare were searched on December 9 2024 for studies involving 1- or 2-level anterior cervical discectomy with quantitative fusion assessment via CT or X-ray. A meta-analysis was conducted using a random-effects model to pool fusion rates and their 95% confidence intervals (CIs) at different follow-up points, for different cage types and different cut-off values evaluating fusion. Sixty-four included studies evaluated 5633 patients. Pooled fusion rates increased over time: 55.6% (95% CI: 43.5%, 67.2%) of patients demonstrated fusion at three months, 74.4% (67.6%, 80.1%) at six months, 88.1% at 12 months (85.1%, 90.6%), and 91.8% (89.1%, 93.9%) at 24 months. Subgroup analysis revealed variation in fusion rates depending on cage type, with titanium cages yielding slightly higher rates at all follow-up times. Sensitivity analysis with fusion criteria showed that a cut-off value < 2 mm for interspinous distance yielded lower fusion rates than the cut-off < 2° for Cobb angle at 6 (70% vs. 77.3%), 12 (83.9% vs. 91.1%) and 24 months (89.5% vs. 91.7%). Results on the correlation between fusion and clinical outcomes were inconsistent. Fusion rates improved over time, approaching 56% at 3 months and 90% at 12 months. Notably, to alleviate heterogeneity across studies, there is a dire need to harmonize reporting guidelines in future research.

**Supplementary Information:**

The online version contains supplementary material available at 10.1007/s10143-025-03542-w.

## Clinical trial number

Not applicable.

## Introduction

Anterior cervical discectomy and fusion (ACDF) is a frequently utilized surgical intervention [1–5] in degenerative spine disease to decompress neural tissue. In case of cervical radiculopathy and/or myelopathy, the herniated intervertebral disc is removed via an anterior approach to decompress the nerve root and/or the medulla. In addition to the decompression, an intervertebral device is placed to endorse surgical fusion of the two adjacent vertebrae. Finally, the cage can optionally be secured with anterior plating [1]. Various intervertebral devices are employed as disc replacements to maintain foraminal height and facilitate bony fusion [3, 5].

Literature reports significant variability in the rates of bony fusion following ACDF, a discrepancy largely due to the lack of uniform radiographic standards for evaluating bony fusion [6]. Fusion has commonly been defined as the presence of trabecular bridging on lateral radiographs or CT scans and/or the absence of motion on flexion/extension radiographs. However, these assessments are largely qualitative and subject to expert interpretation. Quantitative assessments of fusion on lateral flexion/extension X-rays include measuring the change in Cobb angle on the operated level and assessing the difference in distance between the ends of the spinous processes. In contemporary practice, there has been a lack of consensus on the fusion assessment methods. While surgical re-exploration might offer the most reliable confirmation of fusion, it is impractical, and even in symptomatic patients, it is preferable to make a diagnosis prior to reoperation [7, 8].

The exact timing at which bony fusion actually is accomplished after ACDF remains uncertain. Although symptoms do not always correlate with the presence of solid fusion, [8, 9] confirming solid fusion, or at least stability, in the targeted motion segment can guide patient activities and potentially explain persistent symptoms [10, 11] To avoid possible complications after surgery, patients’ daily activities are restricted until stability and/or bony fusion is achieved [10] This underscores the importance and relevance of knowing the timing of fusion occurrence. Additionally, fusion rates are also described to be depending on the type of intervertebral device used [11] Enhanced understanding of the influence of different device types on fusion rates could better guide surgeons in selecting the most appropriate device material for surgical procedures [12].

Therefore, the primary objective of this systematic review and meta-analysis was to provide a comprehensive overview of fusion assessment methods and to determine the percentage fusion achieved at different follow-up points. Secondary objectives encompassed investigating the different cut-off values used to evaluate bone fusion, providing an overview of fusion rates associated with different intervertebral devices used, and evaluating the reported correlation between fusion and clinical outcome.

## Materials and methods

### Literature search

This systematic review of the literature was conducted in accordance with the Preferred Reporting Items for Systematic Reviews and Meta-Analyses (PRISMA) checklist [13]. A comprehensive search was conducted in PubMed, Medline, Embase, Web of Science, the Cochrane Library and Emcare to gather all relevant literature. Appropriate Medical Subject Heading (MeSH) terms, title [ti] and text words [tw] were used in the PubMed search (Appendix 1). The search spanned until December 9, 2024.

### Eligibility criteria and study selection

Following the PRISMA checklist, three authors independently screened titles, abstracts and ultimately full-text articles based on predefined in- and exclusion criteria. These were stated as follows: the inclusion criteria for the study required the article to be published in English or Dutch and to present original primary data. The study needed to include a minimum of 10 patients and focus on the cervical spine (C2-Th1). It needed to involve patients undergoing a 1- or 2-level anterior cervical discectomy and fusion with a cage or no intervertebral device (excluding prostheses). Fusion measurement had to be conducted using a CT scan or dynamic X-ray, supplying a quantitative outcome, and a follow-up period of at least three months. The method for assessing fusion needed to be described, and the article had to be published in a peer-reviewed journal. The exclusion criteria specified that the study should not include patients with instability in the posterior cervical spine elements, patients with additional fusion of the posterior spinal column, or patients undergoing anterior discectomy due to a traumatic lesion, bone disease, or malignancy. Additionally, fusion assessment should not be based solely on expert opinion.

Any disagreements between the authors were resolved by discussion. If needed, a senior reviewer was consulted.

### Risk of bias assessment

Three reviewers independently assessed the quality of the selected studies using an adjusted version of the Dutch Cochrane Centre checklist for cohort studies, as detailed in Appendix 2 [10]. Each study arm of a study was evaluated separately as a case series, as our focus was solely on the fusion rates within each study arm rather than the differences between groups. Each study was evaluated for selection bias, outcome bias, and follow-up bias, with each category contributing a maximum of 3 points. Consequently, a study could receive a total score of up to 9 points. Based on their scores, studies were categorized into either a low risk of bias group (5–9 points) or a high risk of bias group (4 or fewer points) using a method adapted from Furlan et al. [14]. Any disagreements were discussed and resolved amongst the authors; if consensus was not reached, a senior author gave a final judgement.

For outcomes with at least 10 studies, potential small study effects were assessed using a funnel plot to visually detect asymmetry around the pooled point estimate and Begg’s test to determine statistical significance [15]. When small study effects were detected and heterogeneity was low, the trim-and-fill method was employed to estimate and include the potentially missing studies, followed by recalculating the pooled point estimate, contingent on small study effects being the only source of asymmetry. When heterogeneity was high, the fail-safe N method, which is the number of studies with non-significant results that would need to be added to drive the total result to non-significance, was employed instead.

### Data extraction

Relevant data from each included study were extracted (if available) by three independent authors as follows: (1) study characteristics, (2) baseline demographics (sample size, age, gender, follow-up time), (3) method of measuring bony fusion, (4) fusion rates at 3, 6, 12, 24, 36, 48 and 60 months (%), (5) different cage types used, (6) correlation between fusion and clinical outcomes.

### Data analysis

We only included studies providing fusion rates at one of the pre-determined follow-up times (3, 6, 12, 24, 36, 48 and 60 months) in our main analysis. Fusion rates at all these follow-up times were pooled utilizing a random-effects model, which was calculated via the DerSimonian and Laird method [16]. At all timepoints, sub-group analyses were conducted based on different cage types used. To reduce heterogeneity, sensitivity analyses were conducted to assess the fusion rate for only the studies that used the cut-off value < 2° for Cobb angle measurements or studies that used < 2 mm for interspinous distance measurements. The presence of heterogeneity was assessed using Cochrane Q statistics with a significance level of *p* < 0.10 [17]. The degree of heterogeneity among studies was determined using Thompson’s I² value, reported as low, medium, and high with I² values of 25%, 50%, and 75%, respectively [18, 19]. Unless otherwise specified, a two-sided p-value of < 0.05 was considered statistically significant. The analysis was performed in Comprehensive Meta-Analysis version 4.

## Results

**Fig. 1:**
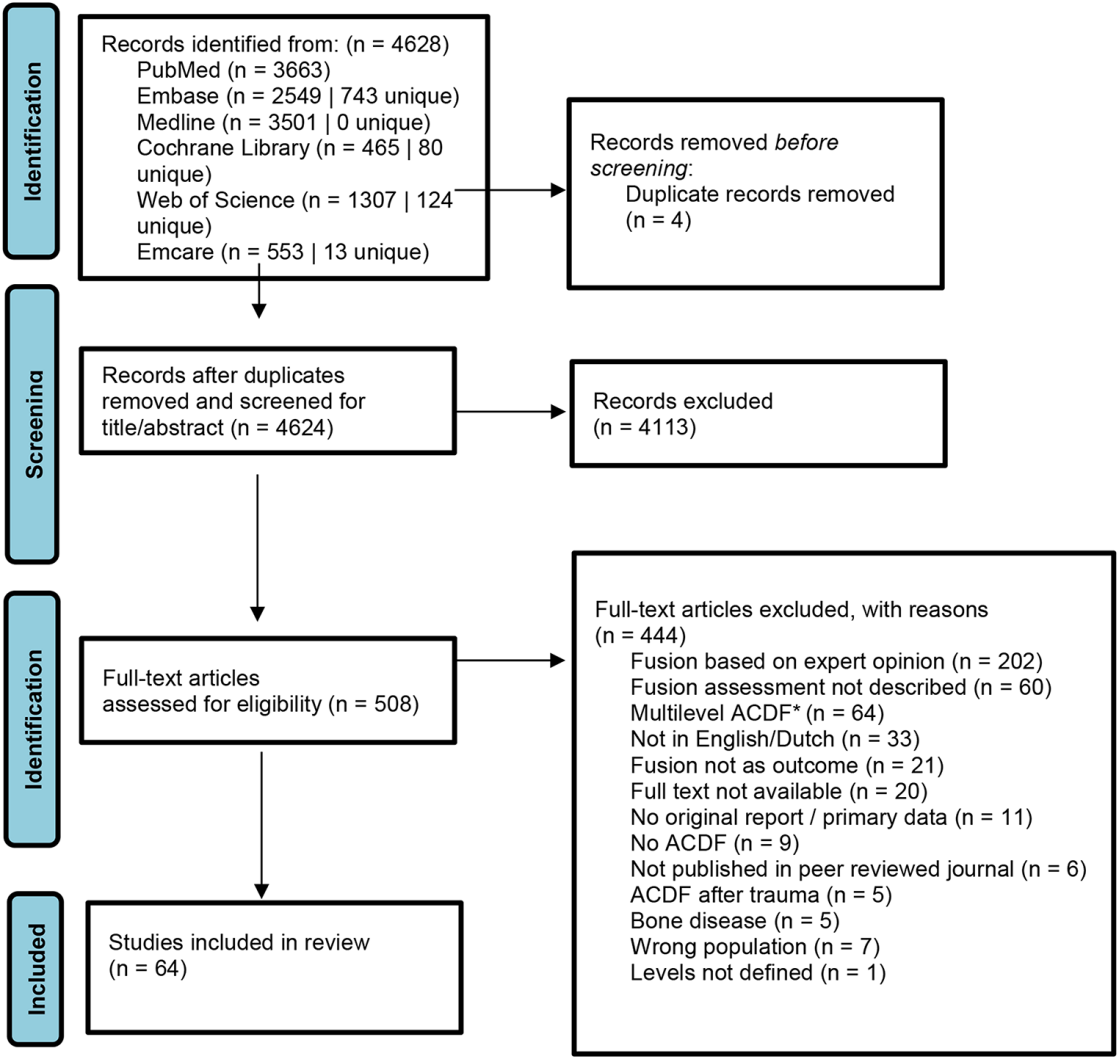
Prisma flowchart *anterior cervical discectomy and fusion

A total of 4628 articles were retrieved from this search. Of those, 4113 were excluded in title and abstract screening. After full text review, 444 studies were excluded as shown in Fig. 1.

Sixty-four unique studies, encompassing 5633 patients were eligible for the systematic review [5, 20–75]. Studies reporting fusion rates at the pre-determined follow-up times were pooled. Out of the 64 studies included, 43 were retrospective cohort studies, 10 were prospective cohort studies and 11 were randomized controlled trials. In the meta-analysis, each study-arm of each study was treated as a case series, as we are not investigating differences between study-arms, but bony fusion within each individual group. This resulted in a total of 112 case series out of these 64 studies. Twelve studies, comprising 23 case series, reported fusion rates at 3 months. Twenty-three studies, consisting of 40 case series, reported fusion rates at 6 months. Thirty-six studies, including 63 case series, reported fusion rates at 12 months of FU. Thirteen studies, comprising 24 case series, reported fusion rates at 24 months of FU. At 36, 48, and 60 months, only two studies, consisting of three case series, reported fusion rates (Appendix 3).

### Risk of bias assessment

A total of 49 studies were assessed to have a low risk of bias, and 15 studies showed a high risk of bias.

### Distribution of cages over patients

**Fig. 2:**
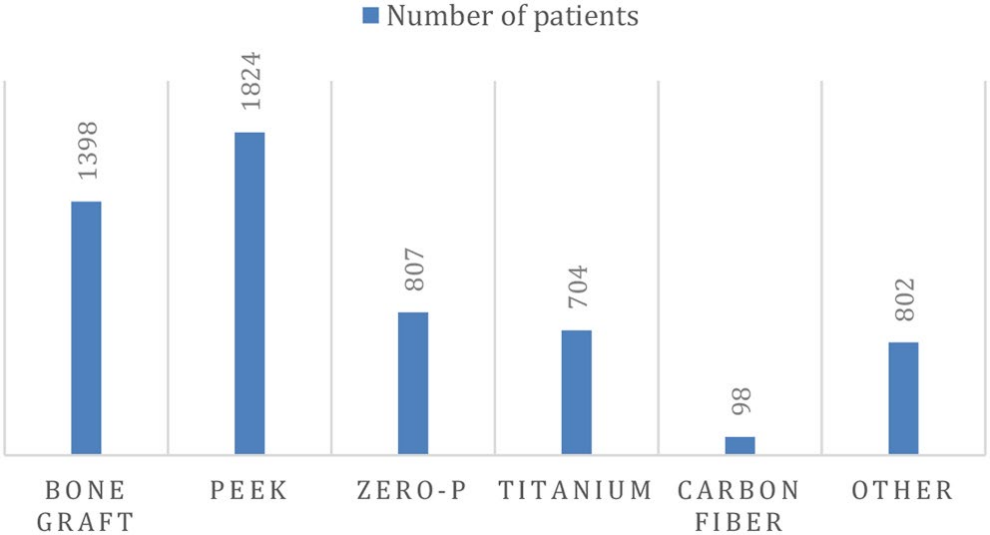
Distribution of patients that underwent anterior discectomy over cage types

Combining all studies resulted in a cohort of 5633 patients, of whom 1398 received a bone graft (including allogenic and autologous bone), 1824 received a polyetheretherketone (PEEK) cage, 807 received a Zero-P cage, 704 received a titanium cage and 98 received a carbon fiber cage (Fig. 2).

### Methods of measuring bony fusion

All studies quantified bony fusion using lateral flexion/extension X-rays, and none supplied a quantitative CT evaluation. In 31 studies, the angulation changes (Cobb angle) between flexion/extension at the target level were measured; in 22 studies, the difference in interspinous distances upon flexion and extension was measured; and in 10 studies fusion was assessed measuring the Cobb angle and the interspinous distance. The cut-off points in angulation changes (in degrees) and differences in interspinous distances (in mm) on flexion/extension X-rays varied among articles (Table 1). An overview of the fusion assessment method used in each study is shown in Appendix 3.

**Table 1:** Overview of cut-off values for measuring fusion using flexion/extension radiographs

Cut-off levels	Studies (*n*)
5°	4
4°	6
3°	1
2°	30
3 mm	1
2 mm	17
1 mm	12
Other^1^	3

^1^Other: Cobb angle: 10°, Interspinous distance: 0032 mm; 1.25 mm

### Fusion rates

#### Fusion rates at three months

**Fig. 3:**
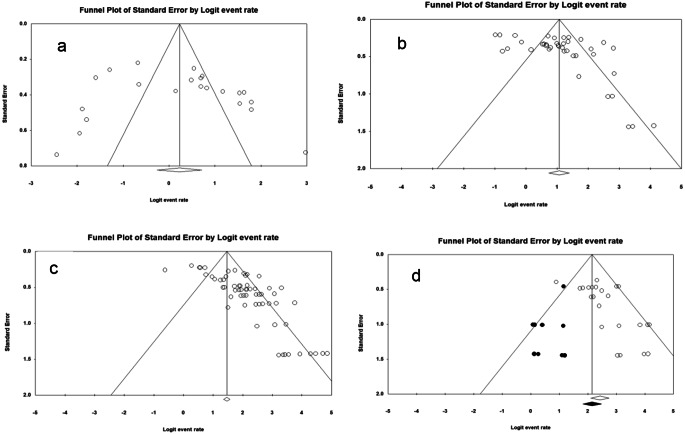
Funnel Plot of Standard Error by Logit event rate at **(a)** 3-month follow-up; **(b)** 6-month follow-up; **(c)** 12-month follow-up; and **(d)** 24-month follow-up. The vertical solid line indicates the pooled logit fusion rate, and the two additional lines represent the expected 95% confidence interval for a given standard error. The clear dots represent the individual studies. The center of the clear diamond represents the pooled point estimate on the logit scale. When an asymmetry was observed, the trim-and-fill method was performed when the heterogeneity was low (**d**), while the fail-safe-n was used when the heterogeneity was high (**b** and **c**)

A total of 12 studies consisting of 23 case series reported the fusion rates at the 3-month follow-up. [20, 22, 23, 31, 37, 38, 39, 47, 50, 62, 69, 71. The overall fusion rate was determined to be 55.6% (95% CI: 43.5–67.2%), meaning about 56% of the patients demonstrated fusion. The analysis indicated high heterogeneity (I²: 90.9%; p-heterogeneity: 0.000). There was no evidence of small study effects based on the symmetry observed in the funnel plot and the Begg’s test (p-value: 0.15) (Fig. 3a). Excluding the low quality study did not materially change the fusion rate or heterogeneity [47], as the overall fusion rate was 53.2% (95% CI: 41.2–64.9%) (I²: 90.7%; p-heterogeneity: 0.000).

#### Fusion rates at six months

A total of 23 studies consisting of 40 case series reported fusion rates at the six-month follow-up [5, 20, 21, 22, 23, 26, 27, 31, 32, 36, 39, 40, 41, 50, 59, 60, 62, 66, 68, 69, 71, 76, 77]. The overall fusion rate was calculated to be 74.4% (95% CI: 67.6–80.1%). The analysis showed high heterogeneity (I²: 88.4%; p-heterogeneity: 0.000). There was an evidence of a potential small study effects based on the asymmetry in the funnel plot and the Begg’s test (p-value: 0.02) (Fig. 3b). The fail-safe N analysis indicated that 2604 additional studies with null results would be required to render the meta-analysis of the 40 included studies non-significant. This number is suggesting a low potential vulnerability to publication bias. Excluding the low quality study did not materially change the fusion rates or heterogeneity [68], as the overall fusion rate was 73.8% (95% CI: 66.9–79.7%) (I²: 88.3%; p-heterogeneity: 0.000).

#### Fusion rates at 12 months

A total of 36 studies consisting of 63 case series reported fusion rates at the twelve-month follow-up [5, 20–24, 26, 31–34, 36, 39, 41, 46, 50, 55, 56, 58–63, 66, 68–71, 74−80]. The overall fusion rate was calculated to be 88.1% (95% CI: 85.1–90.6%). The analysis indicated high heterogeneity (I²: 79.5%; p-heterogeneity: 0.000). There was an evidence of a potential small study effects based on the asymmetry in the funnel plot and the Begg’s test (p-value < 0.001) (Fig. 3c). The fail-safe N analysis indicated that 3810 additional studies with null results would be required to render the meta-analysis of these 63 studies non-significant. This number is suggesting a low potential vulnerability to publication bias. Excluding low quality studies [24, 33, 34, 64, 68, 70, 75] did not materially change the fusion rate, but did reduce heterogeneity, as the overall fusion rate was 88.6% (95% CI: 86.2–90.7%) (I²: 60.9%; p-heterogeneity: 0.000).

#### Fusion rates at 24 months

A total of thirteen studies, consisting of 24 case series, reported fusion rates at the 24 months follow-up [21, 22, 26, 32, 36, 41, 42, 45, 46, 55, 77, 81, 82]. The overall fusion rate was determined to be 91.8% (95% CI: 89.1–93.9%). The analysis yielded a p-heterogeneity of 0.055 and an I² value of 33.8%, indicating low heterogeneity. Although the funnel plot looked asymmetrical, Begg’s test was not (p-value: 0.12) (Fig. 3d). Contingent on publication bias being the source of asymmetry, the trim-and-fill method revealed a pooled incidence of 89.6% (95% CI: 86.1–92.3%) after having imputed nine potentially missing studies. Excluding the low quality study did not materially change the fusion rate or heterogeneity [82], as the overall fusion rate was 91.8% (95% CI: 88.9–94.0%) (I²: 34.7%; p-heterogeneity: 0.057).

#### Fusion rates at 36 months

Three case series from a total of two high quality studies using bone graft cages reported fusion rates at the 36-month follow-up [21, 43]. The overall fusion rate was determined to be 86.9% (95% CI: 82.1–90.5%). High heterogeneity was indicated by the analysis, which produced an I^2^ value of 89.1% and a p-heterogeneity of 0.000.

#### Fusion rates at 48 months

Two high quality studies using bone graft cages, comprising three case series, reported fusion rates at the 48-month follow-up [21, 29]. The overall fusion rate was found to be 90.9% (95% CI: 85.4–94.5%). The analysis produced a p-heterogeneity of 0.001 and an I² value of 86.1%, indicating high heterogeneity.

#### Fusion rates at 60 months

Two studies with high quality utilizing bone graft cages, consisting of three case series, reported fusion rates at the 60-month follow-up [21, 43]. It was discovered that the total fusion rate was 95.7% (95% CI: 82.1–90.5%). With a p-heterogeneity of 0.076 and an I^2^ value of 61.1%, the study revealed medium heterogeneity.

### Sensitivity analysis of pooled fusion rate by fusion assessment criteria at each evaluation time

The pooled fusion rates for studies that used the cut-off value < 2° for Cobb angle measurements or studies that used the cut-off value < 2 mm for interspinous distance for fusion assessment are presented in Table 2. A cut-off value < 2 mm for interspinous distance resulted in lower fusion rates at each of the 6-, 12- and 24-month follow-up than the cut-off < 2° for Cobb angle. Excluding studies with low quality did not materially change the fusion rates or heterogeneity at 3-, 6- and 24- month follow-up, but heterogeneity slightly decreased at the 12-month timepoint (2°: I^2^ from 6.5 to 0.0%) and (2 mm: I^2^ from 76.5 to 66.7%) (Appendix 4).

**Table 2:** Pooled fusion rates for only the studies with cut-off values < 2° Cobb angle or < 2 mm interspinous distance for fusion assessment, at each follow-up timepoint

Cut-off value	Follow-up (months)	# of case series	Fusion rate (95% CI)	I^2^	*p*- heterogeneity
*Studies of high and low quality*					
< 2°	3	14	53.2% (95% CI: 36.3–69.4%)	91.5%	0.00
	6	18	77.3% (95% CI: 69.2–83.7%)	83.6%	0.00
	12	21	91.1% (95% CI: 88.9–92.9%)	6.5%	0.38
	24	10	91.7% (95% CI: 87.3–94.6%)	27.7%	0.19
< 2 mm	3	5	64.8% (95% CI: 57.8–71.2%)	5.6%	0.38
	6	12	70.0% (95% CI: 60.3–78.2%)	76.6%	0.00
	12	18	83.9% (95% CI: 77.3–88.8%)	76.5%	0.00
	24	5	89.5% (95% CI: 82.2–94.0%)	0.0%	0.70

### Subgroup analysis of pooled fusion rate by cage type at each evaluation time

The pooled fusion rate for the titanium cages was slightly higher at each follow-up time point, after performing the subgroup analyses by cage type (Table 3). In most of the stratified categories, the heterogeneity was reduced as indicated by the I² index and p-heterogeneity. After excluding low quality studies, the fusion rates did not materially change, except for the carbon fiber fusion rate at 12-month follow-up (from 89.5 to 79.5%). Heterogeneity did decrease at three months (58.3–0.0%) and twelve months (84.6–12.5%) in the PEEK cage group and at twelve months (39.4–30.7%) in the titanium group (Appendix 5). In the few categories where the heterogeneity persisted, this could be due to other sources of variation such as the use of different cut-off points to assess fusion, among others.

**Table 3:** Pooled fusion rates for different materials at each follow-up timepoint for all studies that reported on cage type

*Fusion rates for different cage materials at 3-month follow-up:*				
	# of case series	Fusion rate (95% CI)	I^2^	p-heterogeneity
Bone graft	2	82.3% (95% CI: 72.3–89.2%)	0.00%	0.99
PEEK	5	73.9% (95% CI: 62.7–82.6%)	58.30%	0.05
Titanium	1	83.7% (95% CI: 70.6–91.6%)	NA	NA
Zero-P	12	38.6% (95% CI: 24.1–55.5%)	90.50%	0

*Fusion rates for different cage materials at 6-month follow-up:*				
	# of case series	Fusion rate (95% CI)	I^2^	p-heterogeneity
Bone graft	10	64.2% (95% CI: 46.5–78.7%)	91.20%	0
PEEK	7	74.1% (95% CI: 68.4–79.0%)	0.00%	0.82
Titanium	5	92.1% (95% CI: 89.0–94.4%)	0.00%	0.5
Zero-P	13	68.9% (95% CI: 59.4–77.0%)	77.30%	0
Carbon fiber	1	64.1% (95% CI: 48.1–77.5%)	NA	NA

*Fusion rates for different cage materials at 12-month follow-up:*				
	# of case series	Fusion rate (95% CI)	I^2^	p-heterogeneity
Bone graft	15	83.7% (95% CI: 77.0–88.8%)	74.00%	0
PEEK	15	84.4% (95% CI: 75.0–90.7%)	84.60%	0
Titanium	7	92.9% (95% CI: 87.7–96.0%)	39.40%	0.13
Zero-P	13	91.2% (95% CI: 88.3–93.5%)	0.00%	0.47
Carbon fiber	2	89.5% (95% CI: 60.1–98.0%)	78.60%	0.03

*Fusion rates for different cage materials at 24-month follow-up:*				
	# of case series	Fusion rate (95% CI)	I^2^	p-heterogeneity
Bone graft	9	90.9% (95% CI: 84.6–94.7%)	59.80%	0.01
PEEK	6	90.4% (95% CI: 85.5–93.8%)	0.00%	0.83
Titanium	2	98.4% (95% CI: 93.9–99.6%)	0.00%	0.96
Zero-P	2	95.5% (95% CI: 80.3–99.1%)	0.00%	0.36

### Correlation between fusion and clinical outcomes

Five studies have reported on the correlation between fusion and clinical outcomes [30, 46, 47, 53, 72]. According to Lee CH et al., patients demonstrated fusion if the interspinous distance change at the arthrodesis level was ≤ 1 mm. They reported no significant differences between fusion and no-fusion groups in VAS scores for neck pain, arm/shoulder pain, or NDI scores at 24 months (*p* = 0.562, *p* = 0.562, and *p* = 0.779). Lee DH et al. [47] defined fusion as an interspinous distance change ≤ 1 mm and found that improvements in VAS scores for arm pain at one and two years postoperatively were not significantly different between the fusion and no-fusion groups. [46]. However, patients with persistent pseudarthrosis at two-year follow-up demonstrated significantly higher VAS scores for neck pain and NDI scores at one-year postoperatively compared to the final union group (*p* < 0.05). Yoo et al. defined fusion as an interspinous distance change < 2 mm and found no correlation between pseudarthrosis and clinical outcomes, including pain and disability scores [72]. In contrast, Obermueller et al. observed a weak but significant positive correlation (*r* = 0.04, *p* = 0.035) between clinical improvement in VAS scores and fusion as defined by a ≤ 4° Cobb angle [53]. However, fusion defined by other parameters (≤ 2 mm interspinous distance or ≤ 2° Cobb angle) showed no significant correlation. Lastly, De Leo-Vargas et al. defined fusion as movement < 2° Cobb angle and reported no significant differences in NDI scores or VAS pain scores between fusion and no-fusion groups [30].

## Discussion

This systematic review illustrates the complexity of assessing fusion post-ACDF and underscores the necessity for continued advancements in assessment methodologies and how fusion rates are influenced by different cage types. The evolving understanding of how different variables affect fusion rates and how fusion correlates with clinical outcomes is valuable for patient counseling and for enhancing patient outcomes in spinal surgery.

This comprehensive analysis shed light on the variety of approaches used to evaluate fusion after anterior cervical discectomy and fusion (ACDF), highlighting a notable dependence on dynamic imaging modalities like lateral flexion/extension X-rays.

Prior research has demonstrated that static X-ray imaging is generally less accurate in detecting fusion, yielding lower rates of fusion detection. While CT scans provide more detailed imaging, their interpretation remains subjective, preventing them from being considered a gold standard. Dynamic X-rays are frequently employed to assess fusion by measuring interspinous distances and angulation changes, particularly the Cobb angle, as indicators of vertebral stability post-surgery; this indicates a move toward quantitative dynamic assessment modalities for more understanding of vertebral stability after surgery. The review identified that the most commonly used diagnostic criteria for assessing fusion were the 2° Cobb angle (used in 40% of the reviewed case series) and the 2 mm interspinous distance (used in 23% of the reviewed case series). The lack of established criteria in the field is reflected by the diversity in cut-off values for these metrics, which impacts the interpretation of fusion results across various studies. This variability underscores the need for a universally applicable quantitative fusion assessment method [10]. A recent study has established a diagnostic criterion for determining bony fusion, namely the combination of ≤ 3° Cobb angle and ≤ 2 mm interspinous distance (de Vries-Vleggeert criterium) [83]. This prompted us to update our previously published systematic review [10] and conduct a meta-analysis, using this parameter as the primary outcome measure to enable a more accurate conclusion. The established criteria highlight the importance of measuring both the Cobb angle and the interspinous distance when determining fusion. Additionally, these criteria, with an ICC of 0.95, emphasize the need to account for a margin of error in the measurements.

Early fusion rates at three months after ACDF varied greatly, according to the meta-analysis, with a pooled rate of roughly 56%. This variety continued throughout the subsequent follow-up times. Fusion rates generally increased over time, reaching a peak of 88.1% at 12 months and 91.8% at 24 months. These findings show a clear chronological pattern in bone healing after surgery, with significant improvements noted as the recovery time increases. Because of overlapping 95% CIs, the 12- and 24-month fusion rates did not vary statistically. This implies that beyond one-year post-surgery, there are little variations in fusion rates. These results can be used by the physician to predict for the patient when they can resume daily activities. After three months, just over half (55.6%) will be stable, after six months, three-quarters (74.4%), after 12 months, 88.1%, and after 24 months, 91.8%.

Our previously published systematic review [10] showed that at three-month follow-up, fusion had occurred in 51.1% of patients, in 78.3% at six months, in 87.6% at 12 months, and in 92.6% at 24 months. Overall, our results were comparable, but in this updated version our goal was to substantiate them more robustly using an new established diagnostic criterion [83]. However, the literature still shows a significant variation regarding the criteria for determining fusion, presumably leading to substantial heterogeneity in the results. Using the recently established diagnostic criterion, we aimed to incorporate the quality of the fusion assessment in these articles in this update. However, as only one of the studies [76] used this criterium, we performed a sensitivity analysis with the most commonly used diagnostic criteria for fusion assessment (< 2° Cobb angle and < 2 mm interspinous distance) separately, to potentially reduce heterogeneity.

The impact of various cage materials on fusion rates was further defined by this research. Materials such as bone grafts, PEEK cages, titanium cages, and Zero-P devices, showed varying fusion rates, with titanium cages showing a trend for higher fusion rates across multiple follow-up periods. However, given the variability within each cage type itself, these results should be interpreted with caution. It is remarkable that the Zero-P cage performs poorly at the 3 months, given the fact that the cage is secured with screws. However, this difference is no longer present from the 6-month FU onwards.

Results on the correlation between bony fusion and clinical outcomes were inconsistent. While Lee DH et al. [46] identified worse outcomes in patients with persistent pseudarthrosis, Yoo et al. [72] and De Leo-Vargas et al.^30^ found no significant association between fusion and clinical outcomes, including VAS and NDI scores. This variability may be partly attributed to the variety of fusion assessment methods, which ranged from Cobb angle and interspinous distance measurements, all using different cut-off values. Notably, Obermueller et al. [53] reported a weak correlation between fusion and clinical improvement, suggesting that fusion may contribute to improved outcomes. Although fusion is a primary objective in ACDF, its direct impact on clinical outcomes appears to vary among patients and remains unclear. Future research should assess clinical conditions in relation to bony fusion, given our ultimate interest in the patient’s clinical state and its relation with fusion.

The limitations of this study include those inherent with systematic reviews and meta-analyses, as those are restricted solely to data presented in available literature. The heterogeneity reported in this meta-analysis, especially at earlier follow-up periods, points to diverse surgical techniques, devices used, cages sizes, clinical indication, patient populations, and assessment criteria variability used across studies. However, by adhering to strict in- and exclusion criteria, using robust statistical techniques including subgroup analysis by cage type at different follow-up times, and conducting sensitivity analyses by different cut-off points used to assess fusion and by excluding low-quality studies from all of our analyses, heterogeneity was alleviated in most of the subgroups or categories but not in all. The analysis highlighted the necessity for more consistent reporting guidelines and study designs to enhance the comparability and generalizability of findings in future research. Therefore, we recommend to use the de Vries-Vleggeert criteria to assess stability after anterior discectomy surgery in the cervical spine. [83] Additionally, although the review encompassed studies of lower evidence levels and those for which the primary clinical outcome was not specifically bony fusion rate, the results did not materially change after excluding low quality studies. Furthermore, additional variables that might affect fusion rates, such as smoking, steroid usage, and bone density, were not uniformly reported in the studies discussed. Similarly, information on the features of implants, such as their size or surface area available for fusion were not consistently detailed. Consequently, it was not feasible to analyze the impact of these factors on fusion outcomes in this review. Lastly, another limitation is the lack of studies employing time-to-event data, such as survival analyses including hazard ratios. We recommend that future research incorporate survival analysis to more accurately assess time-to-fusion and account for loss to follow-up, in addition to a proper adjustment for confounding.

Notwithstanding, this study had several strengths. By including only articles that utilized quantitative measures for fusion, rather than relying on qualitative assessments based on an expert’s opinion, we aimed to enhance the validity of the results. Additionally, we conducted subgroup analyses based on cage type at each follow-up time, sensitivity analyses based on fusion assessment criteria and by excluding low quality studies to reduce heterogeneity and improve the clinical applicability of the results.

## Conclusion

This systematic review and meta-analysis clarified the challenges in evaluating fusion following ACDF. The findings revealed that fusion rates gradually increased over follow-up time, with significant variability influenced by the type of implant used, notwithstanding a conclusion that in 90% of the patients, stability was achieved after one-year follow-up. This meta-analysis underscores the need for more uniform reporting standards across studies to enhance the reliability and comparability of future research findings, for which we suggest the de Vries-Vleggeert score. Reporting on important variables (smoking, steroid usage, and bone density) as well as features of implants is also needed. Additionally, the relationship between fusion and clinical outcomes remains unclear, indicating a need for further research focusing on this aspect to better understand and improve patient care post-ACDF.

## Electronic supplementary material

Below is the link to the electronic supplementary material.

Supplementary Material 1

## Data Availability

No datasets were generated or analysed during the current study.
