# Supplementary material for: Evaluation of bony fusion after anterior cervical discectomy: a systematic literature review and meta-analysis
Source: Neurosurg Rev. 2025 Apr 25;48(1):386. doi: 10.1007/s10143-025-03542-w (PMC12021957; doi:10.1007/s10143-025-03542-w)
Supplement: Supplementary file 1 — Supplementary Material 1 [file 10143_2025_3542_MOESM1_ESM.pdf]

Appendix 1: Search strategy

PubMed

(("Diskectomy"[Mesh] OR "discectomy"[tw] OR "diskectomy"[tw] OR discectom*[tw] OR diskectom*[tw] OR "ACDF"[tw] OR "Intervertebral Disc Displacement/surgery"[majr]) AND ("Cervical Vertebrae"[Mesh] OR "cervical spine"[tw] OR "Cervical Vertebrae"[tw] OR "Axis"[tw] OR "Odontoid Process"[tw] OR "Cervical Atlas"[tw] OR "Cervical"[tw]) AND ("Prosthesis Implantation"[Mesh] OR "Prostheses and Implants"[Mesh] OR "prosthesis"[tw] OR "prosthetic"[tw] OR "prostheses"[tw] OR "prosthetics"[tw] OR prosthe*[tw] OR "cage"[tw] OR "Spinal Fusion"[Mesh] OR "Spinal Fusion"[tw] OR "implant"[tw] OR "implants"[tw] OR "implantation"[tw] OR "interbody"[tw] OR "spacer"[tw]) NOT ("Animals"[mesh] NOT "Humans"[mesh])) NOT (("Case Reports"[ptyp] OR "case report"[ti] OR "case rep"[all fields]) NOT ("Review"[ptyp] OR "review"[ti] OR "systematic"[sb] OR "Clinical Study"[ptyp] OR "trial"[ti] OR "RCT"[ti] OR "randomized"[ti] OR "randomised"[ti] OR "random*"[ti]))
Search date: 9 December 2024 and #hits: 3663

MEDLINE via OVID

(exp "Diskectomy"/ OR "discectomy".mp OR "diskectomy".mp OR discectom*.mp OR diskectom*.mp OR "ACDF".mp) AND (exp "Cervical Vertebrae"/ OR "cervical spine".mp OR "Cervical Vertebrae".mp OR "Axis".mp OR "Odontoid Process".mp OR "Cervical Atlas".mp OR "Cervical".mp) AND (exp "Prosthesis Implantation"/ OR exp "Prostheses and Implants"/ OR "prosthesis".mp OR "prosthetic".mp OR "prostheses".mp OR "prosthetics".mp OR "prosthe*".mp OR "cage".mp OR exp "Spinal Fusion"/ OR "Spinal Fusion".mp OR "implant".mp OR "implants".mp OR "implantation".mp OR "interbody".mp OR "spacer".mp) NOT (exp "Animals"/ NOT exp "Humans"/) NOT ((exp "Case Reports"/ OR "case report".ti OR "case rep".af OR (case and reports).jw OR (case and report).jw) NOT (exp "Review"/ OR "review".ti OR exp "Systematic Review"/ OR exp "Clinical Study"/ OR "trial".ti OR "RCT".ti OR "randomized".ti OR "randomised".ti OR "random*".ti))
Search date: 9 December 2024 and #hits: 3501 (0 unique)

Embase

((*"intervertebral diskectomy"/ OR "discectomy".ti OR "diskectomy".ti OR "discectom*".ti OR "diskectom*".ti OR "ACDF".ti OR *"intervertebral disk hernia"/su) AND (exp "Cervical Spine"/ OR "cervical spine".ti,ab OR "Cervical Vertebrae".ti,ab OR "Axis".ti,ab OR "Odontoid Process".ti,ab OR "Cervical Atlas".ti,ab OR "Cervical".ti,ab) AND (exp "Prosthesis"/ OR "Implantation"/ OR "prosthesis".ti,ab OR "prosthetic".ti,ab OR "prostheses".ti,ab OR "prosthetics".ti,ab OR "prosthe*".ti,ab OR "cage".ti,ab OR exp "Spinal Fusion"/ OR "Spinal Fusion".ti,ab OR "implant".ti,ab OR "implants".ti,ab OR "implantation".ti,ab OR "interbody".ti,ab OR "spacer".ti,ab) NOT (exp "Animals"/ NOT exp "Humans"/)) NOT ((exp "Case Report"/ OR "case report".ti OR "case rep".af OR (case and reports).jx OR (case and report).jx) NOT (exp

"Review"/ OR "review".ti OR exp "Systematic Review"/ OR exp "Clinical Study"/ OR "trial".ti OR "RCT".ti OR "randomized".ti OR "randomised".ti OR "random*".ti))

- NOT (conference review or conference abstract).pt

Search date: 9 December 2024 and #hits: 2549 (743 unique)

Web of Science

(((TI=("intervertebral diskectomy" OR "discectomy" OR "diskectomy" OR discectom* OR diskectom* OR "ACDF") OR AK=("intervertebral diskectomy" OR "discectomy" OR "diskectomy" OR discectom* OR diskectom* OR "ACDF")) AND TS=("Cervical Spine" OR "cervical spine" OR "Cervical Vertebrae" OR "Odontoid Process" OR "Cervical Atlas" OR "Cervical") AND TS=("Prosthesis" OR "Implantation" OR "prosthesis" OR "prosthetic" OR "prostheses" OR "prosthetics" OR prosthe* OR "cage" OR "Spinal Fusion" OR "Spinal Fusion" OR "implant" OR "implants" OR "implantation" OR "interbody" OR "spacer") NOT TI=("veterinary" OR "rabbit" OR "rabbits" OR "animal" OR "animals" OR "mouse" OR "mice" OR "rodent" OR "rodents" OR "rat" OR "rats" OR "pig" OR "pigs" OR "porcine" OR "horse" OR "horses" OR "equine" OR "cow" OR "cows" OR "bovine" OR "goat" OR "goats" OR "sheep" OR "ovine" OR "canine" OR "dog" OR "dogs" OR "feline" OR "cat" OR "cats")) NOT (TI=("Case Report" OR "case report") OR AK=("Case Report" OR "case report") OR SO=("Case Report*"))

Search date: 9 December 2024 and #hits: 1307 (124 unique)

Cochrane

((("intervertebral diskectomy" OR "discectomy" OR "diskectomy" OR discectom* OR diskectom* OR "ACDF") AND ("Cervical Spine" OR "cervical spine" OR "Cervical Vertebrae" OR "Axis" OR "Odontoid Process" OR "Cervical Atlas" OR "Cervical") AND ("Prosthesis" OR "Implantation" OR "prosthesis" OR "prosthetic" OR "prostheses" OR "prosthetics" OR prosthe* OR "cage" OR "Spinal Fusion" OR "Spinal Fusion" OR "implant" OR "implants" OR "implantation" OR "interbody" OR "spacer")):ti,ab,kw

NOT (conference abstract OR meeting abstract OR conference proceeding OR conference proceedings):pt

Search date: 9 December 2024 and #hits: 465 (80 unique)

Emcare

((*"intervertebral diskectomy"/ OR "discectomy".ti OR "diskectomy".ti OR "discectom*".ti OR "diskectom*".ti OR "ACDF".ti) AND (exp "Cervical Spine"/ OR "cervical spine".ti,ab OR "Cervical Vertebrae".ti,ab OR "Axis".ti,ab OR "Odontoid Process".ti,ab OR "Cervical Atlas".ti,ab OR "Cervical".ti,ab) AND (exp "Prosthesis"/ OR "Implantation"/ OR "prosthesis".ti,ab OR "prosthetic".ti,ab OR "prostheses".ti,ab OR "prosthetics".ti,ab OR "prosthe*".ti,ab OR "cage".ti,ab OR exp "Spinal Fusion"/ OR "Spinal Fusion".ti,ab OR "implant".ti,ab OR "implants".ti,ab OR "implantation".ti,ab OR "interbody".ti,ab OR "spacer".ti,ab) NOT (exp "Animals"/ NOT exp "Humans"/)) NOT ((exp "Case Report"/ OR "case report".ti OR "case rep".af OR (case and reports).jx OR (case and report).jx) NOT (exp "Review"/ OR "review".ti OR exp "Systematic Review"/ OR exp "Clinical Study"/ OR "trial".ti OR "RCT".ti OR "randomized".ti OR "randomised".ti OR "random*".ti))

Search date: 9 December 2024 and #hits: 553 (13 unique)

Appendix 2: Quality assessment checklist according to an adjusted version of the Dutch Cochrane Centre checklist.

	Award 1 point if
Selection bias (3 pts.)	
Goal and inclusion	Goal of the study is stated and study explicitly states the inclusion criteria
Selection of patients	Selective recruitment of patients can be ruled out
Patient characteristics	Study reports the age range and mean age and states the distribution of gender
Outcome bias (3 pts.)	
Definition of fusion	Definition classification and radiological tools to measure fusion were stated
Clinical outcome	Clinical outcome was systematically evaluated in correlation with fusion
Radiographic method	Fusion was measured through CT scan
Follow-up bias (3 pts.)	
Follow-up time frame	Follow-up range, period and mean were given and loss to follow-up <20%
Prospective study	Data in the study were collected prospectively
Multiple moments	Follow-up was divided into multiple moments in time
Total (9 pts.)	

Appendix 3: Patients' characteristics of the studies included in the meta-analysis

Author and year†	Follow-up (months)	Cage type	Fusion criteria	Fusion rate (%) | evaluation time	Patients (n)	Risk of Bias‡
Hacker R, 2000a	6, 12, 24	Titanium cage noncoated (BAK-C)	Less than 2° of segmental movement on lateral F/E views and no more than 50% radiolucency covering the implant's outer surfaces	92.4% | 6 months<br>92.8% | 12 months<br>98.4% | 24 months	179	6 (low)
Hacker R, 2000b	6, 12, 24	Titanium cage with hydroxyapatite (HA-BAK-C)	Less than 2° of segmental movement on lateral F/E views and no more than 50% radiolucency covering the implant's outer surfaces	94.4% | 6 months<br>96.5% | 12 months<br>98.5% | 24 months	167	6 (low)
Hacker R, 2000c	6, 12, 24	Iliac crest autograft / allograft	Less than 2° of segmental movement on lateral F/E views and no more than 50% radiolucency covering the implant's outer surfaces	79.8% | 6 months<br>88.6% | 12 months<br>90.8% | 24 months	142	6 (low)
Assietti R, 2002	12	Carbon fiber cage + bone chips/biphasic calcium phosphate	Segmental motion of less than 2°, endplates had disappeared into both adjacent VBs and if the two VBs formed a block, with no radiolucency demonstrated except by the cage itself	96%	24	3 (high)
Zevgaridis D, 2002a	12	Iliac crest autograft	Less than 2° of segmental movement on lateral flexion–extension views	88.8%	18	5 (low)
Zevgaridis D, 2002b	12	Titanium	Less than 2° of segmental movement on lateral flexion–extension views	83.3%	18	5 (low)

Baskin D, 2003a	6, 12, 24	Fibular allograft + INFUSE bone graft (RhBMP-2)	Less than 4° angular motion on flexion-extension X-rays, no radiolucency and evidence of bridging trabecular bone	100% | 6 months<br>100% | 12 months<br>100% | 24 months	18	6 (low)
Baskin D, 2003b	6, 12, 24	Fibular allograft + Autogenous iliac crest bone graft	Less than 4° angular motion on flexion-extension X-rays, no radiolucency and evidence of bridging trabecular bone	100% | 6 months<br>100% | 12 months<br>100% | 24 months	15	6 (low)
Türeyen K, 2003	18.0 (mean)	Titanium cage	Less than 2˚ of segmental motion	98%	43	4 (high)
Goldberg G, 2007a	10 (mean)	Autologous iliac crest bone graft	Quantitative motion analysis: Motion threshold of 2° used to define a solid fusion	76.2%	21	3 (high)
Goldberg G, 2007b	9.5 (mean)	Fibular allograft bone	Quantitative motion analysis: Motion threshold of 2° used to define a solid fusion	81.8%	22	3 (high)
Fernandez-Fairen M, 2008a	6, 12, 24	Porous tantalum cage (standalone)	Evidence of bony bridging around the implant and/or 2° of variation of Cobb’s angle on F/E radiographs or 2 mm of variation in the interspinous distance, in the absence of periimplant radiolucency	82.1% | 6 months<br>89.3% | 12 months<br>89.3% | 24 months	28	7 (low)
Fernandez-Fairen M, 2008b	6, 12, 14	Autograft (autologous tricortical bone)	Evidence of bony bridging around the implant and/or 2° of variation of Cobb’s angle on F/E radiographs or 2 mm of variation in the interspinous	78.7% | 6 months<br>84.4% | 12 months<br>84.4% | 24 months	33	7 (low)

			distance, in the absence of periimplant radiolucency			
Song K, 2009a	30.5 (mean)	PEEK cage	Fusion was defined as (1) less than 2° movement on lateral flexion/extension views	94.1%	17	4 (high)
Song K, 2009b	29.9 (mean)	PEEK cage	Fusion was defined as (1) less than 2° movement on lateral flexion/extension views	66.7%	21	4 (high)
Song K, 2009c	31.1 (mean)	PEEK + CSLP cage	Fusion was defined as (1) less than 2° movement on lateral flexion/extension views	100%	18	4 (high)
Song K, 2009d	29.9 (mean)	PEEK + CSLP cage	Fusion was defined as (1) less than 2° movement on lateral flexion/extension views	95.5%	22	4 (high)
Wang X, 2009	6, 12	Titanium BAK-C with autograft (iliac)	Segmental movement was <2 degrees on extension-flexion radiographs	89.1% | 6 months<br>100% | 12 months	64	3 (high)
Lee CH, 2013a	6, 12	Autogenous iliac tricortical bone block and plate (IP)	- X-ray: Fusion was defined as a distance change of <2 mm between the tips of the spinous processes at the surgically treated level, as measured from the flexion-extension lateral radiographs	70% | 6 months<br>87.1% | 12 months	32	6 (low)
Lee CH, 2013b	6, 12	Installation of both a carbon	- X-ray: Fusion was defined as a distance change of <2 mm between the	64.1% | 6 months<br>79.5% | 12 months	39	6 (low)

		fiber compisate cage and a plate (auto/allograft)	tips of the spinous processes at the surgically treated level, as measured from the flexion-extension lateral radiographs			
Lee CH, 2013c	6, 12	Cancellous (auto/allograft) bonefilled solis cage without plating	- X-ray: Fusion was defined as a distance change of <2 mm between the tips of the spinous processes at the surgically treated level, as measured from the flexion-extension lateral radiographs	41.6% | 6 months<br>63.2% | 12 months	87	6 (low)
Kasliwal M, 2013	6, 12, 24	Iliac crest autograft	Patients were deemed fused if no differences in angulation (<2 degrees) or the alteration of interspinous process distance (<2 mm) was observed	90.0% | 6 months<br>100% | 12 months<br>100% | 24 months	11	6 (low)
Oh J, 2013a	23.4 (mean)	PEEK cage alone	Fusion was defined as <2-degree movement on lateral flexion/extension views, the presence of bridging trabecular bone between the endplates on anteroposterior/ lateral views, the lack of implant failure signs of the anterior plate system, and <50% radiolucency in the perimeter surrounding the cage	96.4%	28	5 (low)
Oh J, 2013b	20.6 (mean)	PEEK cage + plate	Fusion was defined as <2-degree movement on lateral flexion/extension views, the presence of bridging trabecular bone between the endplates	96.2%	26	5 (low)

			on anteroposterior/ lateral views, the lack of implant failure signs of the anterior plate system, and <50% radiolucency in the perimeter surrounding the cage			
Park JH, 2013a	12, 24	(PEEK) cage filled with PolyBone	Fusion criteria included stability on dynamic view (motion between the adjacent spinous processes º2 mm) and evidence of formation of a contiguous bony bridge on lateral radiographs and CT scans	79.2% | 12 months<br>91.7% | 24 months	24	7 (low)
Park JH, 2013b	12, 24	(PEEK) cage filled with iliac bone	Fusion criteria included stability on dynamic view (motion between the adjacent spinous processes º2 mm) and evidence of formation of a contiguous bony bridge on lateral radiographs and CT scans	95.7% | 12 months<br>95.7% | 24 months	23	7 (low)
Wang HR, 2013	43,6 (mean)	PEEK cage standalone self-locking	Absence of movement of >2 mm between the spinous processes on flexion extension lateral x-rays.	93.9%	16	5 (low)
Yoo M, 2014a	24 (mean)	CFCF (carbon fiber composite frame cages)	Fusion was defined as a distance change of less than 2 mm between the tips of the spinous processes of the surgically treated level based on flexion-extension lateral radiographs	68.8%	35	4 (high)
Yoo M, 2014b	24 (mean)	PEEK cage	Fusion was defined as a distance change of less than 2 mm between the	82.6%	23	4 (high)

			tips of the spinous processes of the surgically treated level based on flexion-extension lateral radiographs			
Chang H, 2015a	21.1 (mean)	Autograft	Difference of < 2 degrees between flexed and extended lateral radiographs, (2) formation of a bony bridge between two endplates, (3) no findings of implant failure, and (4) radiolucency in < 50% of the tissue around the implant	100%	38	2 (high)
Chang H, 2015b	19.3 (mean)	Allograft	Difference of < 2 degrees between flexed and extended lateral radiographs, (2) formation of a bony bridge between two endplates, (3) no findings of implant failure, and (4) radiolucency in < 50% of the tissue around the implant	95.5%	44	2 (high)
Chang H, 2015c	18.5 (mean)	Zero-P	Difference of < 2 degrees between flexed and extended lateral radiographs, (2) formation of a bony bridge between two endplates, (3) no findings of implant failure, and (4) radiolucency in < 50% of the tissue around the implant	95.8%	48	2 (high)
Davis R, 2015	48	Cancellous autograft	Less than 2° of angular motion in flexion/extension and evidence of bridging bone across the disc space	85.2%	105	5 (low)

			and radiolucent lines at no more than 50% of the graft vertebral interfaces			
Luo J, 2015	36.9 (mean)	PEEK cage	X-ray: (1) the absence of motion >2 mm between the spinous processes on flexion– extension lateral radiographs; (2) the absence of a radiolucent gap between the graft and the endplate; and (3) the presence of continuous bridging trabeculae at the graft and endplate junction	100%	26	5 (low)
Marbacher S, 2015	61.14 (mean)	Stand-alone PCTC titanium cages	A level was interpreted as fused when bridging bony trabeculae were seen and when there was < 2 degrees of motion	88%	33	5 (low)
Yi J, 2015a	3, 6, 12	Hydroxyapacite (HA) + DBM	A difference in ISDs greater than 2 mm was defined as a nonunion	69.0% | 3 months<br>76.3% | 6 months<br>86.8% | 12 months	38	7 (low)
Yi J, 2015b	3, 6, 12	Hydroxyapacite (HA) + β-TCP	A difference in ISDs greater than 2 mm was defined as a nonunion	74.0% | 3 months<br>74.0% | 6 months<br>87.1% | 12 months	39	7 (low)
Gok H, 2016	12	PEEK cage (empty bladed)	Bridging bone inside or outside of the cage on CT, and if there was < 2° of segmental motion on flexion / extension radiographs	92%	25	4 (high)

Park JY, 2016a	21.31 (mean)	PEEK cage + DBM and local bone chips	If there was less than two degrees of motion at the fusion site or less than 2 mm gap in the interspinous distance on the flexion and extension radiographs, stability was assumed	96.2%	26	5 (low)
Park JY, 2016b	16.42 (mean)	PEEK cage + DBM and local bone chips	If there was less than two degrees of motion at the fusion site or less than 2 mm gap in the interspinous distance on the flexion and extension radiographs, stability was assumed	92.2%	51	5 (low)
Qizhi S, 2016	6, 12	Zero-P	Solid fusion of the involved segments was achieved <2° on extension-flexion radiographs	100%	17	6 (low)
Vanichkachorn J, 2016	6,12	PEEK cage	Equal or less than 4 degrees angular motion from flexion/extension X-rays	78.6% | 6 months<br>93.5% | 12 months	31	8 (low)
Arts M, 2017a	3, 6, 12, 24	Silicon nitride spacer	Rotation ≤4° and ≤1.25 mm translation on flexion–extension films	66.7% | 3 months<br>76.3% | 6 months<br>81.4% | 12 months<br>93% | 24 months	52	7 (low)
Arts M, 2017b	3, 6, 12, 24	PEEK cage	Rotation ≤4° and ≤1.25 mm translation on flexion–extension films	67.6% | 3 months<br>72.5% | 6 months<br>90.2% | 12 months<br>90.2% | 24 months	48	7 (low)
Bucci M, 2017	6	ROI-C zeroP PEEK +	Bony bridging with less than 2° segmental motion in flexion/extension and less than 3 mm AP translation	85.7%	38	6 (low)

		titanium coated + autograft bone				
Kim S, 2017a	24	PEEK cage with demineralized bone matrix	<2° movement on the lateral flexion/ extension views, the presence of bridging trabecular bone be tween the endplates on antero-posterior/lateral views, and <50% radiolucency on the perimeter surrounding the cage	86.1%	36	5 (low)
Kim S, 2017b	24	PEEK cage with demineralized bone matrix	<2° movement on the lateral flexion/ extension views, the presence of bridging trabecular bone be tween the endplates on antero-posterior/lateral views, and <50% radiolucency on the perimeter surrounding the cage	93.8%	48	5 (low)
Lanman T, 2017	36, 60	Cortical ring allograft	Angulation of target level ≤4° of angular motion, based on lateral flexion/extension radiographs & evidence of bridging bone, based on the evidence of a continuous bony connection w/ VBs above & below in at least 1 of the following areas: lateral, anterior, posterior, &/or through the allograft ring, & no evidence of radiolucency covering >50% of either the superior or inferior surface of the graft	83.3% | 36 months<br>94.0% | 60 months	188	6 (low)
Peppers T, 2017	6, 12	PEEK cage packed with TE	≤4°angular motion on flexion/extension plain radiographs.	89.4%	40	7 (low)

		= cellular bone allograft				
Yu J, 2018a	36.9 (mean)	ACDF with PEEK standalone cages and plate fixation	Movement less than 2° and widening of the interspinous distance of <2 mm on lateral flexion and extension views	97.3%	76	5 (low)
Yu J, 2018b	39.1 (mean)	ACDF with PEEK standalone cages	Movement less than 2° and widening of the interspinous distance of <2 mm on lateral flexion and extension views	96.3%	82	5 (low)
Yu J, 2018c	38.5 (mean)	ACDF with iliac bone and plate fixation	Movement less than 2° and widening of the interspinous distance of <2 mm on lateral flexion and extension views	97.7%	89	5 (low)
Lee DH, 2018	12, 24	Freeze-dried fibular cortical ring allograft filled with local bone	1) the interspinous distance change of more than 1 mm on 150% or more magnified flexion/extension lateral x-ray; (2) the absence of bridging bone across the graft into the adjacent endplates and/or bridging bone outside of the graft; or (3) the radio- lucent lines extending more than 50% from the cortical-host bone interface on x-ray or CT scan	67.4% | 12 months<br>91% | 24 months	89	6 (low)
Mastronardi L, 2018	3,6,12	Standalone trabecular tantalum metal	The flexion-extension range of motion was 5 degrees	21.6% | 3 months<br>68.2% | 6 months<br>97.7% | 12 months	88	6 (low)

Noh S, 2018a	32.7 (mean)	Perfect-C (PEEK)	Radiological fusion was defined that there was ≤ 2° motion and/or ≤ 2 mm of motion of the interspinous distance on flexion–extension x-rays	90%	41	5 (low)
Noh S, 2018b	32.7 (mean_	Zero-P	Radiological fusion was defined that there was ≤ 2° motion and/or ≤ 2 mm of motion of the interspinous distance on flexion–extension x-rays	86%	36	5 (low)
Noh S, 2018c	32.7 (mean	PEEK plate + cage	Radiological fusion was defined that there was ≤ 2° motion and/or ≤ 2 mm of motion of the interspinous distance on flexion–extension x-rays	95%	71	5 (low)
De Leo-Vargas R, 2019	6.7 (mean)	Zero-P (standalone) + DBX	Movement <2° in postoperative flexion-extension radiographs, (2) presence of trabeculae bridging bone formation at the anterior and/or posterior cortex of the involved vertebral bodies and absence of radiolucency through the fusion levels	95.8%	53	5 (low)
Lee CH, 2019	3	PEEK cage filled with biphasic calcium phosphate ceramic	Fusion was characterized by <2° movement through lateral flexion–extension radiography, bridging bone between end- plates, no signs of implant failure of PEEK cage–plate system, and <50% of radiolucency covering the implant's outer surface	95.1%	41	4 (high)

Arts M, 2020a	3, 6, 12	Porous titanium (additional bone graft)	Rotation ≤4° and ≤1.25 mm translation on flexion–extension films	84% | 3 months<br>89% | 6 months<br>91% | 12 months	49	7 (low)
Arts M, 2020b	3, 6, 12	PEEK cage + autograft	Rotation ≤4° and ≤1.25 mm translation on flexion–extension films	67% | 3 months<br>72% | 6 months<br>90% | 12 months	48	7 (low)
Lee CJ, 2020	24	Autologous bone graft	The plain radiographic ISM criteria for fusion at the arthrodesis level was defined as ISM <1 mm, and at a non-arthrodesed superjacent level was defined as ISM > 4 mm, based on 150% magnified dynamic radiographs	71%	31	5 (low)
Huang K, 2020a	3, 6, 12	Zero-P	1) less than 2°of segmental movement on lateral flexion/ extension views.	33.7% | 3 months<br>77.2% | 6 months<br>89.1% | 12 months	92	6 (low)
Huang K, 2020b	3, 6 ,12	Zero-P	1) less than 2°of segmental movement on lateral flexion/ extension views.	12.5% | 3 months<br>54.2% | 6 months<br>87.5% | 12 months	24	6 (low)
Obermueller T, 2020	29.99 (mean)	PEEK + DBM<br>PEEK without DBM	ISM < 2 mm	58%	208	6 (low)
Park S, 2020a	12	Bioactive glass ceramic (BGC)	ISM < 1 mm	88.5%	26	7 (low)
Park S, 2020b	12	Allograft	ISM < 1 mm	82.9%	37	7 (low)

Abudouaini H, 2021a	3, 6, and 12	Zero P + b-tricalcium phosphate	≤2° motion and/or ≤ 2 mm of motion of the interspinous distance on flexion-extension X-rays	61.9% | 3 months<br>73.8% | 6 months<br>90.5% | 12 months	42	6 (low)
Abudouaini H, 2021b	3, 6, 12	Zero P + b-tricalcium phosphate	≤2° motion and/or ≤ 2 mm of motion of the interspinous distance on flexion-extension X-rays	63.2% | 3 months<br>79.4% | 6 months<br>95.6% | 12 months	68	6 (low)
Abudouaini H, 2021c	3, 6, 12	Zero P + b-tricalcium phosphate	≤2° motion and/or ≤ 2 mm of motion of the interspinous distance on flexion-extension X-rays	53.6% | 3 months<br>67.7% | 6 months<br>92.7% | 12 months	28	6 (low)
He S, 2021a	3	Intervertebral cage (removed bone from patient)	a) adjacent vertebral displacement was <2° in flexion with neck extension; b) target cervical intervertebral space height remained unchanged; c) no transparent lines were detected between the grafted bone and the upper and lower vertebral body endplates; d) it is judged as subsidence if the height of intervertebral space is more than 3 mm	85.7%	45	6 (low)
He S, 2021b	3	Zero-P ROI-C spacer	a) adjacent vertebral displacement was <2° in flexion with neck extension; b) target cervical intervertebral space height remained unchanged; c) no transparent lines were detected between the grafted bone and the upper and lower vertebral body endplates; d) it is judged as subsidence	82.2%	42	6 (low)

			if the height of intervertebral space is more than 3 mm			
Jin Y, 2021	6	3D-printed porous titanium interbody fusion cage	X-ray taken in the cervical flexion-extension dynamic position showed that the angle of the fusion intervertebral body changed by <5 2. No obvious translucent bands around the fuser or displacement were detected. 3. The titanium plate screw system did not loosen, and the surrounding bone transparent area was <50% of its surrounding surface	100%	30	7 (low)
Noh S, 2021a	39.1 (mean)	Zero - P plate + spacer device	Radiologic fusion was defined when there was <2 degrees of flexion–extension movement and/or when there was <0032 mm of movement of the interspinous process upon flexion–extension across the fusion segment	95%	38	5 (low)
Noh S, 2021b	39.1 (mean)	PEEK cage + plate	Radiologic fusion was defined when there was <2 degrees of flexion–extension movement and/or when there was <0032 mm of movement of the interspinous process upon flexion–extension across the fusion segment	94%	42	5 (low)
Quek C, 2021	12	PEEK/allograft	Absence of motion of > 2 mm between the spinous processes on flexion-extension lateral radiographs	82%	90	5 (low)

Wang XJ, 2021a	3, 6, 12	Zero-P + beta-tricalcium	Lack of evidence of > 2° motion	34.2% | 3 months<br>63.2% | 6 months<br>92.1% | 12 months	38	6<br>(low)
Wang XJ, 2021b	3, 6, 12	Zero-P + beta-tricalcium	Lack of evidence of > 2° motion	14.3% | 3 months<br>35.7% | 6 months<br>89.3% | 12 months	28	6<br>(low)
Wang XJ, 2021c	3, 6, 12	Zero-P + beta-tricalcium	Lack of evidence of > 2° motion	8.0% | 3 months<br>32.0% | 6 months<br>80.0% | 12 months	25	6<br>(low)
Yang JJ, 2021a	12	Allograft (corticocancellous)	Interspinous motion (ISM) <2 mm on a 150% magnified flexion/extension lateral radiograph	74.3%	35	4<br>(high)
Yang JJ, 2021b	12	Allograft (corticocancellous)	Interspinous motion (ISM) <2 mm on a 150% magnified flexion/extension lateral radiograph	68.1%	44	4<br>(high)
Godlewski B, 2022	12	Interbody implant + nanoparticle hydroxyapatite	<2 mm change between tips of spinous processes in flexion/extension	56.7%	104	4<br>(high)
He S, 2022a	3	Zero-P ROI-C spacer	(i) adjacent vertebral displacement <2 in extension and flexion of neck; (ii) unaltered intervertebral space (IVS) height; and (iii) absence of a transparent line between the grafted bone and the top and bottom vertebral endplates	85.7%	35	6<br>(low)

He S, 2022b	3	Intervertebral cage (removed bone from patient)	(i) adjacent vertebral displacement <2 in extension and flexion of neck; (ii) unaltered intervertebral space (IVS) height; and (iii) absence of a transparent line between the grafted bone and the top and bottom vertebral endplates	82.4%	34	6 (low)
Lawless M, 2022	17.9 (mean)	PEEK cage	X-ray, Fusion was recorded if there was 1 mm or less of interspinous motion at the operative segment when magnified to 150% and with 4 mm or more of superjacent interspinous motion above the operative segment	79.5%	301	5 (low)
Zhang J, 2022a	12	Cage plate fixation	Less than 10° of motion on flexion/extension films;	97%	33	2 (high)
Zhang J, 2022b	12	Zero-P	Less than 10° of motion on flexion/extension films;	100%	35	2 (high)
Arnold P, 2023a	6, 12, 24, 36, 48, 60	Allograft ring + I-factor	X-ray evidence of bridging trabecular bone between the involved motion segments, translational motion <3 mm and angular motion <5°	30% | 6 months<br>89.5% | 12 months<br>95.5% | 24 months<br>99% | 36,48,60 months	106	6 (low)
Arnold P, 2023b	6, 12 ,24, 36, 48, 60	Allograft ring + local autograft	X-ray evidence of bridging trabecular bone between the involved motion segments, translational motion <3 mm and angular motion <5°	27.5% | 6 months<br>85% | 12 months<br>96% | 24 months	114	6 (low)

				98.2% | 36,48,60 months		
Atici Y, 2023a	40.5 (mean)	Titanium cage + autograft	Bridging trabecular bone between the end plates, absence of a radiolucent gap between the graft and the end plate, and <1 mm of motion between the spinous processes during extension and flexion	94.1%	17	4 (high)
Atici Y, 2023b	37.4 (mean)	Titanium cage + autograft	Bridging trabecular bone between the end plates, absence of a radiolucent gap between the graft and the end plate, and <1 mm of motion between the spinous processes during extension and flexion	100%	25	4 (high)
Deng Y, 2023	3, 6, 12	Zero-P	No more than 2° of angular motion on dynamic radiographs; 2) the absence of radiolucent gap between the grafts and endplates; 3) the presence of continuous bridging bony trabeculae at graft–endplate interface	16.9% | 3 months<br>71.2% | 6 months<br>94.8% | 12 months	77	6 (low)
Lee JJ, 2023a	24	Anchored cage	<2° in the lateral flexion/extension view, the presence of the bridging trabecular bone between the endplates in the anteroposterior/ lateral view, and 50% radiation transmittance around the cage perimeter	88%	43	4 (high)

Lee JJ, 2023b	24	Anchored cage	<2° in the lateral flexion/extension view, the presence of the bridging trabecular bone between the endplates in the anteroposterior/ lateral view, and 50% radiation transmittance around the cage perimeter	97%	47	4 (high)
Park J, 2023a	12	PEEK cage + HA and beta-TCP	Fusion was defined as an average interspinous distance of <1 mm on dynamic radiographs at the operated level at 12-month postoperative follow-up	73.7%	40	5 (low)
Park J, 2023b	12	BGS-7 (glass ceramic) interbody spacer	Fusion was defined as an average interspinous distance of <1 mm on dynamic radiographs at the operated level at 12-month postoperative follow-up	78.1%	36	5 (low)
Sheng X, 2023a	3, 6, 12	UJF device with autologous iliac cancellous bone	Dynamic radiography showing that the difference between the upper and the lower spinous processes was less than 1 mm	66.7% | 3 months<br>94.1% | 6 months<br>100% | 12 months	36	7 (low)
Sheng X, 2023b	3, 6, 12	Zero-P + cancellous iliac bone	Dynamic radiography showing that the difference between the upper and the lower spinous processes was less than 1 mm	13.2% | 3 months<br>66.7% | 6 months<br>94.6% | 12 months	38	7 (low)
Song K, 2023a	12	PEEK cage	<1 mm of ISM	35%	66	3 (high)

Song K, 2023b	12	PEEK + plate	<1 mm of ISM	64%	83	3 (high)
Croft A, 2024a	12	PEEK cage	< 1 mm between flexion and extension radiographs, along with superjacent segment interspinous motion ≥ 4 mm	93%	42	6 (low)
Croft A, 2024b	12	Tritanium	< 1 mm between flexion and extension radiographs, along with superjacent segment interspinous motion ≥ 4 mm	82%	11	6 (low)
Goldman S, 2024	6,12	Allogenic cellular bone matrix	Evidence of bridging bone across the disc space on CT, angular motion <3 degrees, and translational motion <2 mm on lateral radiographs.	46.5% | 6 months<br>100% | 12 months	43	6 (low)
Ma Y, 2024a	12	Titanium alloy	Change in intervertebral space angle of fusion segment on cervical dynamic radiograph<5°	100%	25	5 (low)
Ma Y, 2024b	12	PEEK cage	Change in intervertebral space angle of fusion segment on cervical dynamic radiograph<5°	85.2%	27	5 (low)
Shin HK, 2024a	12	Stand-alone cage	1 mm or less of inter spinous motion at the operative segment when magnified to 150% and 4 mm or more of superjacent interspinous motion	94.7%	38	5 (low)
Shin HK, 2024b	12	Stand-alone cage	1 mm or less of inter spinous motion at the operative segment when	93.3%	45	5 (low)

			magnified to 150% and 4 mm or more of superjacent interspinous motion			
Wu P, 2024a	24	Zero-P	<1-mm of interspinous motion on flexion-extension radiographs	100%	30	5 (low)
Wu P, 2024b	24	ROI-C	<1-mm of interspinous motion on flexion-extension radiographs	100%	26	5 (low)
Zhao H, 2024a	6,12,24	Zero-P	Interspinous motion < 1mm	84.6% | 6 months<br>91.3% | 12 months<br>92.3% | 24 months	13	6 (low)
Zhao H, 2024b	6,12,24	PEEK cage	Interspinous motion < 1mm	83.3% | 6 months<br>86.7% | 12 months<br>90% | 24 months	30	6 (low)

†In the meta-analysis, each study-arm of each study was treated as a case series (letters after the year), as we were not investigating differences between study-arms, but bony fusion within each individual group.
‡assessed using an adjusted version of the Dutch Cochrane Centre checklist.

Appendix 4: Pooled fusion rates for only the studies with cut-off values < 2° Cobb angle or < 2 mm interspinous distance for fusion assessment at each follow-up timepoint, excluding low quality studies.

Cut-off value	Follow-up (months)	# of case series	Fusion rate (95% CI)	I^2	p-heterogeneity
Excluding low quality studies					
**< 2°**	3	13	48.7% (95% CI: 32.5% to 65.3%)	91.2%	0.00
	6	17	76.3% (95% CI: 67.8% to 83.2%)	83.8%	0.00
	12	18	90.6% (95% CI: 88.4% to 92.5%)	0.0%	0.47
	24	8	91.6% (95% CI: 86.5% to 94.9%)	28.9%	0.20
**< 2 mm**	3	5	64.8% (95% CI: 57.8% to 71.2%)	5.6%	0.38
	6	12	70.0% (95% CI: 60.3% to 78.2%)	76.6%	0.00
	12	15	87.0% (95% CI: 81.0% to 91.3%)	66.7%	0.00
	24	5	89.5% (95% CI: 82.2% to 94.0%)	0.0%	0.70

Appendix 5. Pooled fusion rates for different materials at each follow-up timepoint for studies that reported on cage type, excluding low quality studies.

Fusion rates for different cage materials at 3-month follow-up:

	# of case series	Fusion rate (95% CI)	I^2	p-heterogeneity
**Bone graft**	2	82.3% (95% CI: 72.3% to 89.2%)	0.0%	0.99
**PEEK**	4	69.2% (95% CI: 61.9% to 75.8%)	0.0%	0.76
**Titanium**	1	83.7% (95% CI: 70.6% to 91.6%)	NA	NA
**Zero-P**	12	38.6% (95% CI: 24.1% to 55.5%)	90.5%	0.00

Fusion rates for different cage materials at 6-month follow-up:

	# of case series	Fusion rate (95% CI)	I^2	p-heterogeneity
**Bone graft**	10	64.2% (95% CI: 46.5% to 78.7%)	91.2%	0.00
**PEEK**	7	74.1% (95% CI: 68.4% to 79.0%)	0.0%	0.82
**Titanium**	4	92.9% (95% CI: 89.5% to 95.2%)	0.0%	0.51
**Zero-P**	13	68.9% (95% CI: 59.4% to 77.0%)	77.3%	0.00
**Carbon fiber**	1	64.1% (95% CI: 48.1% to 77.5%)	NA	NA

Fusion rates for different cage materials at 12-month follow-up:

	# of case series	Fusion rate (95% CI)	I^2	p-heterogeneity
**Bone graft**	13	86.0% (95% CI: 78.8% to 91.1%)	75.9%	0.00
**PEEK**	12	86.6% (95% CI: 82.5% to 89.8%)	12.5%	0.32
**Titanium**	6	92.3% (95% CI: 87.2% to 95.4%)	30.7%	0.21
**Zero-P**	12	91.0% (95% CI: 87.9% to 93.3%)	0.0%	0.63
**Carbon fiber**	1	79.5% (95% CI: 64.0% to 89.4%)	NA	NA

Fusion rates for different cage materials at 24-month follow-up:

	# of case series	Fusion rate (95% CI)	I^2	p-heterogeneity
**Bone graft**	9	90.9% (95% CI: 84.6% to 94.7%)	59.8%	0.01
**PEEK**	6	90.4% (95% CI: 85.5% to 93.8%)	0.0%	0.83
**Titanium**	2	98.4% (95% CI: 93.9% to 99.6%)	0.0%	0.96
**Zero-P**	2	95.5% (95% CI: 80.3% to 99.1%)	0.0%	0.36
